# Distribution of crystalloid fluid infused during onset of anesthesia-induced hypotension: a retrospective population kinetic analysis

**DOI:** 10.1186/s13741-021-00204-5

**Published:** 2021-10-11

**Authors:** Robert G. Hahn

**Affiliations:** 1 Research Unit, Södertälje Hospital, 152 86 Södertälje, Sweden; 2 Karolinska Institutet at Danderyds Hospital (KIDS), Stockholm, Sweden

**Keywords:** Anesthesia, Intravenous, Anesthesia, Regional, Hypotension, Vascular, Pharmacokinetics, Therapy, Fluid

## Abstract

**Background:**

Induction of anesthesia causes a drop in arterial pressure that might change the kinetics of infused crystalloid fluid. The aim of this report is to provide a mathematical view of how fluid distributes in this setting.

**Methods:**

Data were retrieved from three studies where 76 patients (mean age 63 years, mean body weight 66 kg) had received approximately 1.1 L of Ringer’s solution over 60 min by intravenous infusion before and during induction of spinal, epidural, or general anesthesia. A population kinetic model was used to analyze the fluid distribution and its relationship to individual-specific factors. Frequent measurements of blood hemoglobin and the urinary excretion served as dependent variables.

**Results:**

Before anesthesia induction, distribution to the extravascular space was threefold faster than elimination by urinary excretion. Both distribution and elimination of infused fluid were retarded in an exponential fashion due to the anesthesia-induced decrease in the mean arterial pressure (MAP). A decrease in MAP from 110 to 60 mmHg reduced the rate of distribution by 75% and the rate of elimination by 90%. These adaptations cause most of the infused fluid to remain in the bloodstream. Age, gender, type of anesthesia, and the use of ephedrine had no statistically significant effect on plasma volume expansion, apart from their possible influence on MAP.

**Conclusion:**

The decrease in MAP that accompanies anesthesia induction depresses the blood hemoglobin concentration by inhibiting both the distribution and elimination of infused crystalloid fluid. The report provides mathematical information about the degree of these changes.

**Supplementary Information:**

The online version contains supplementary material available at 10.1186/s13741-021-00204-5.

## Introduction

Crystalloid fluid is often administered intravenously (i.v.) during induction of anesthesia (McCrae and Wildsmith 1993), although this fluid is considered to have a poor plasma volume–expanding effect (Jacob et al. 2012). Volume loading before the induction (pre-loading) does not prevent a drop in arterial pressure (Coe and Revenäs, 1990; Ewaldsson and Hahn 2005) while providing the fluid during the onset of anesthesia (co-loading) can reduce the magnitude of the drop, which is best studied in parturients (James and Dyer 2016).

One reason for why co-loading maintains the arterial pressure better than pre-loading might be that more volume remains in the plasma in close connection with the infusion. A larger proportion of infused fluid is known to remain in the plasma when hypotension has just been induced (Hahn 1992). A recent kinetic study in which the fluid was given only after the induction showed an arrested distribution that resolved only 20 min later (Hahn and Nemme 2020), resulting in a plasma volume expansion that temporarily approached 100% of the infused amount. Besides being of physiological interest, the excessive volume expansion is mirrored by a reduction of the blood hemoglobin (Hb) concentration that affects oxygen delivery, the planning of deliberate hemodilution, and estimates of the blood loss allowed before initiating erythrocyte transfusion.

The aim of the present report was to obtain a mathematical analysis of what governs the distribution of crystalloid fluid when given during the onset of anesthesia. The report is based on a population kinetic analysis based on plasma dilution and urinary excretion data derived from studies in which crystalloid fluid was given to clinical patients as continuous infusion before, during, and after the onset of spinal, epidural, or general anesthesia. Special consideration was given to the role of the mean arterial pressure (MAP), but other factors, such as gender and the choice of fluid, were also evaluated as having potential importance regarding this aspect of the adaptation to the anesthetized state.

## Methods

This study is a retrospective population kinetic analysis that uses data from three previously published studies of fluid distribution during induction of anesthesia (Ewaldsson and Hahn 2001, 2005; Li et al. 2007). The analysis comprised pooled data from 76 infusions in which lactated or acetated Ringer’s solution had been administered by i.v. infusion in a similar way to patients during induction of spinal, epidural, or general anesthesia. All three studies had been approved by the appropriate Ethics Committee before the first patient was enrolled. Written informed consent was obtained from all patients before starting the study.

### Anesthesia procedures

The infusions were initiated between 7 and 9 AM. The patients had fasted overnight and were placed on a bed to rest for 30 min to reach a hemodynamic steady state. In the first two studies, a cannula was placed in the cubital vein of each arm, one for blood sampling and the other for infusion of fluid. The arm used for blood sampling was placed on a body-warm heating pad. In the third study, arterial blood was sampled.

Anesthesia was usually induced when 1/3 of the infusions had been administered. The types of anesthesia used in the three studies are shown in Table 1. Spinal anesthesia was induced in the left lateral decubitus position using a 25G Whitacre needle. The subarachnoid space was punctured in the L_3_ and L_4_ interspace or, if that was unsuccessful, in the L_2_-L_3_ interspace. Plain bupivacaine (2.4–3.4 mL; Marcain-Spinal, 5 mg/mL; AstraZeneca, Södertälje, Sweden) was injected as required to achieve surgical anesthesia.

**Table 1:** Demographic data for the cohorts used for population volume kinetic analysis

	Ewaldsson and Hahn 2001	Ewaldsson and Hahn 2005	Li et al. 2007
Females/males	1/4	9/11	18/33
Infusions (*N*)	5	20	53
Age (years)	70 (54-80)	65 (33-80)	61 (24-86)
Body weight (kg)	85 (79-106)	76 (54-120)	61 (45-96)
Fluid volume (mL/kg)	15	20	≈17
Infusion time (min)	40	60	60
Infusion rate (mL/min/kg)	0.375	0.333	0.286
Regional/general anesthesia	5/0	9/11	20/33
Type of regional anesthesia	Spinal	Spinal	Epidural
Induction at (min)	20	20	15
Patients given ephedrine (*N*)	1	5	7
Study duration (min)	50	60	60

Data are the mean (range)

Epidural anesthesia was applied through a 17-gauge Tuohy needle with the patient in the same body position. Increments of 3–5 mL 0.5% ropivacaine (Pharmacia, AstraZeneca, Germany) were injected every 5 min until a surgical block was achieved.

General anesthesia was induced with propofol (1.5 mg/kg), midazolam (0.05 mg/kg), sufentanil (0.6 μg/kg), and rocuronium bromide (0.6 mg/kg), and anesthesia was then maintained with propofol (3 mg/kg/h) and rocuronium bromide (4 mg/h).

### Measurements

The plasma volume was expanded by infusing the Ringer solution i.v. using infusion pumps. During and after these infusions, venous blood (3–4 mL) was withdrawn to measure the Hb concentration and the hematocrit (Hct) on the apparatus used for routine measurements in the hospital’s Clinical Chemistry Laboratory. The samples were withdrawn every 3–5 min in a standardized manner to ensure a coefficient of variation (CV) of about 1%. The baseline sample was drawn in duplicate, and the mean of the two concentrations was used in subsequent calculations.

The excreted urine was collected via an indwelling catheter, which had been inserted into the bladder under topical anesthesia before the studies started.

MAP was measured non-invasively with an automatic device (Datex AS3, Datex, Helsinki, Finland) (Ewaldsson and Hahn 2001, 2005), while invasive measurements were performed and displayed on a multifunction Datex-Ohmeda instrument (Hoevelaken, The Netherlands) when an arterial line had been established (Li et al. 2007).

An intravenous bolus dose of 5 mg of ephedrine, which could be repeated if necessary, was given if the systolic arterial pressure fell to 60% of baseline or if the patient experienced near-fainting symptoms (nausea, sweating, and bradycardia). No other vasopressor was used.

### Kinetic analysis

Population (mixed effects) kinetics is an industry-standard tool for evaluating and recommending dosing regimens for drugs with regard to individual-specific factors, such as age, gender, and body weight (Heeremans et al. 2010). The volume kinetic method is a modification of drug pharmacokinetics for the study of infusion fluids, but it differs from conventional pharmacokinetics in that the fluid compartments have expandable walls. A benefit of this approach is that it allows analysis of dynamic events, as this is difficult to achieve with radioactive tracer methods.

Volume kinetics is based on repeated measurement of the blood Hb concentration, which is the inverse of the blood water concentration (Hahn 2020). Infusion fluids contain almost exclusively water; therefore, Hb changes are an index of the water volume that rapidly equilibrates with the circulating blood.

A two-volume kinetic model with micro-constants was simultaneously fitted to all data for the dependent variables, which were the frequently measured plasma dilution and the urinary excretion.

The appropriateness of all fixed parameters was challenged one by one to arrive at an optimal base model. Thereafter, the influence of covariates on the fixed parameters was tested sequentially, as guided by a reduction in the residual error for the model (Owen and Fiedler-Kelly 2014).

#### Base model

In the finally used base model, fluid is infused at rate *R*_o_ to expand the volume of a central body fluid space *V*_c_ to *v*_c_. The volume expansion is written (*v*_c_–*V*_c_).

**Fig. 1:**
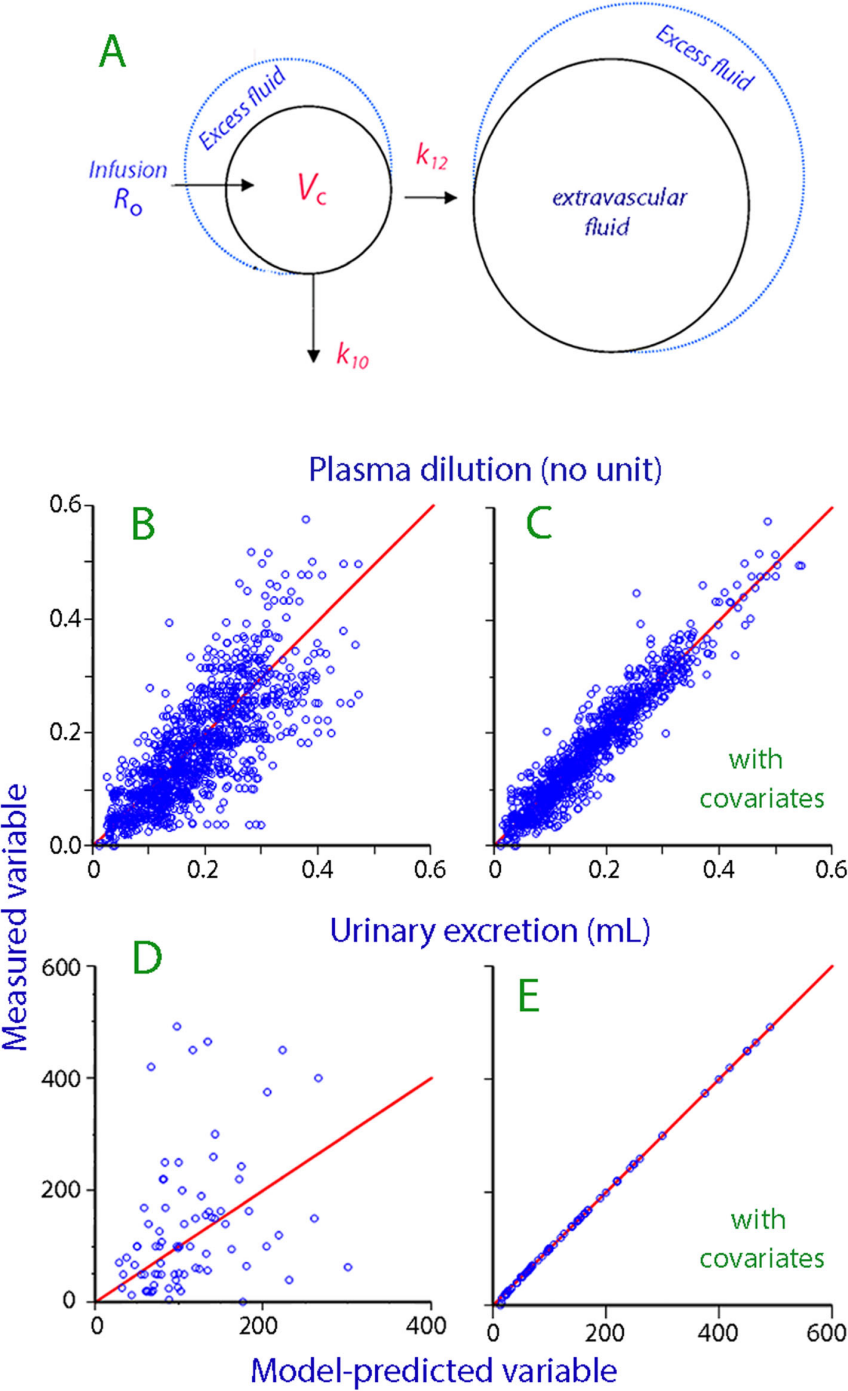
Kinetic model and goodness-of-fit. (**A**) Schematic drawing of the volume kinetic model. (**B**) Predicted versus measured plasma dilution for all data points to the base model. (**C**) Same plot after correction for the covariates. Random distribution around the solid line of unity indicates a good goodness-of-fit. (**D**) Predicted versus measured urinary excretion for all patients according to the base model. (**E**) Same plot after correction for covariates

Distribution of the fluid to a peripheral body fluid space is governed by a rate constant *k*_12_, and the flow from the central to the peripheral space at any time is given by the product of *k*_12_ and (*v*_*c*_–*V*_c_). Similarly, the elimination is the product of the volume expansion of *V*_c_ at any time and an elimination rate constant, *k*_10_ (Fig. 1A).

The differential equations are:

$$ {\displaystyle \begin{array}{l}\mathrm{d}{v}_c/\mathrm{dt}={R}_{\mathrm{o}}-{k}_{12}\left({v}_c-{V}_c\right)-{k}_{10}\left({v}_c-{V}_c\right)\\ {}\mathrm{d}U/ dt={k}_{10}\left({v}_c-{V}_c\right)\end{array}} $$

where *U* is the measured urinary excretion.

The plasma dilution is used to indicate the volume expansion of *V*_c_ resulting from the infusion. Hence:

$$ \left({v}_c-{V}_c\right)/{V}_c=\left[\left(\mathrm{Hb}/\mathrm{hb}\right)-1\right]/\left(1-\mathrm{Hematocrit}\right) $$

Symbols in capital letters denote baseline values. A minor correction is made for the effects of blood sampling on the plasma dilution (Hahn 2010).

#### Covariates

A search for trends in plots of random effects (eta:s) was used to identify covariate candidates. A trend in an eta plot could suggest the existence of a significant covariate. The most promising candidate parameters were then tested, one by one, by adding them to the base model in a diagonal design, which assumes no correlation between random effects. The threshold for accepting one curve-fit as significantly better than another was guided by a reduction of > 3.8 points in the goodness-of-fit (−2 LL = log likelihood) for the model; where > 6.6 points represents *P* < 0.01. The base model with the significant covariates added to it constituted the final model and had the lowest residual error of all runs.

Gender, age, body weight, general or regional anesthesia (spinal/epidural), type of Ringer’s (lactated or acetated), the use of ephedrine, and the rate of infusion per kilo body weight, as well as various time factors, were sequentially tested as covariates to the three fixed parameters in the model. The mean arterial pressure (MAP) and the heart rate were evaluated as time-varying covariates, which mean that a new value was considered for each point of measurement. Both the crude value of MAP and the change in MAP from baseline were tested.

#### Parameter estimation

The fixed parameters in the model and the statistically significant covariates, if any, were estimated simultaneously using the Phoenix software for nonlinear mixed effects (NLME), version 1.3 (Pharsight, St. Louis, MO) with the First-Order Conditional Estimation Extended Least Squares as a search routine and an additive model for the random-error variability (Owen and Fiedler-Kelly 2014). While the covariates were added with a diagonal design, the finally reported parameter values represent a “Full Block Model” that considers correlations between random effects and is considered more accurate for simulation purposes.

The goodness-of-fit of the model was studied by residual plotting, where the dependent variables were recreated from the fixed parameters with and without consideration of the covariates.

The performance of the model was illustrated by predictive checks (1000 iterations) and bootstrap analysis (500 runs, with random sampling and replacement), using the built-in features of the Phoenix software.

Demographic data were reported as the mean (standard deviation), and the kinetic data were reported as the mean (95% confidence interval).

## Results

Table 1 shows the characteristics of the 76 infusion experiments. The patient mean age was 63 (standard deviation, 15) years, the mean body weight was 66 (17) kg, and each patient received 1157 (323) mL of Ringer’s. The kinetic analysis was based on 1198 measurements of plasma dilution and 128 measurements of the urine volume. The original data are given in Supplementary file 1.

### Base model

The search for an optimal base model resulted in a number of modifications of the conventional two-volume model (Fig. 1A). The most important change is that the rate constant *k*_21_ was removed due to lack of statistical significance. Several add-ons were tried that have occasionally been included in previous work. These include a second elimination function and an absorption function, but none of them improved the model. Hence, the variant described in the “Methods” section, with one expandable fluid space (*V*_c_), one distribution (*k*_12_), and one elimination (*k*_10_) function, was found to be optimal. The search strategy used to find the final population kinetic model is shown in Table 2.

**Table 2:** Key features of the search protocol used to build the final population kinetic model. The strategy is to reduce −2(LL) by more than 3.8 points in each step, which means that the change of the model is statistically significant

Optimization routine	Model	Target parameter	LL	−2 (LL)	AIC
Naive pooled	5 parameters (*V*_c_, *k*_12_, *k*_21_, *k*_10_, and *k*_b_)		340	−681	−631
Naive pooled	Removal of first fixed parameter	*k*_b_	341	−681	−669
FOCE ELS	Removal of first fixed parameter	*k*_b_	1280	−2560	−2540
FOCE ELS	Removal of second fixed parameter	*k*_21_	1541	−3081	−3065
FOCE ELS	Add covariate: body weight	*V*_c_	1558	−3117	−3099
	Add covariate: crude MAP	*k*_12_	1605	−3210	−3190
FOCE ELS	Add covariate: crude MAP	*k*_10_	1632	−3264	−3242
FOCE ELS	Full block model	All the above	1652	−3304	−3276
FOCE ELS	Bootstrap analysis	All the above	1652	−3304	−3246

*FOCE ELS* forward conditional extended least squares method, *LL* log likelihood, *AIC* Akaike criterion

### Included covariates

The covariate search showed that the body weight (BW) affected *V*_c_ and that the absolute (crude) value of MAP significantly influenced both *k*_12_ and *k*_10_. The model parameters in the final analysis are shown in Table 3, which explains that the values of the fixed (group) parameters were modified by the body weight and by the individual-specific MAP at each timepoint to create the following unique model parameter for each timepoint of each individual patient:

$$ {\displaystyle \begin{array}{l}{V}_c=2,146\left[{\left(\mathrm{BW}/66\right)}^{0.86}\right]\\ {}{k}_{12}=22.1\;\mathrm{x}\;{10}^{-3}\left[{\left(\mathrm{MAP}/94.4\right)}^{2.34}\right]\\ {}{k}_{10}=5.1\;\mathrm{x}\;{10}^{-3}\left[{\left(\mathrm{MAP}/94.4\right)}^{3.73}\right]\end{array}} $$

**Table 3:** Population kinetic parameters in the final model

	Covariate	Best estimate	95% CI	RSE	Bootstrap 95% CI
Kinetic parameter					
V_c_ (L)		2.15	1.88–2.42	6.4	1.88–2.45
*k*_12_ (10^−3^ min^−1^)		22.1	15.3–28.8	15.6	16.3–29.1
*k*_10_ (10^−3^ min^−1^)		5.1	3.9–6.4	12.1	4.0–6.5
Covariate effects					
V_c_	Body weight	0.86	0.51–1.21	19.8	0.51–1.16
*k*_12_	MAP	2.34	1.87–2.82	10.4	1.65–3.36
*k*_10_	MAP	3.73	1.99–5.47	23.7	2.31–5.74

*V*_c_ central volume of distribution; conversion factor between plasma dilution and plasma volume expansion, *k*_12_ rate constant for translocation of fluid from *V*_c_ to the extravascular fluid space, *k*_10_ rate constant governing urinary excretion, *CI* confidence interval, *RSE* standard error/best estimate

where 66 is the mean patient body weight and 94.4 is the mean MAP for all 1198 observations. The MAP at baseline was 107.3 (standard deviation, 9.3) mmHg.

### Discarded covariates

Several potential covariates were tested that did not reach statistical significance. These included age; gender; ratio and change of MAP from baseline; the use of ephedrine (*n* = 13); use of regional/general anesthesia; and the time period before versus after the induction. Acetated Ringer’s seemed to distribute more rapidly than lactated Ringer’s, but this difference lost significance in the final model.

In accordance with a previous study (Hahn and Nemme 2020), the distribution (*k*_12_) was hypothesized to be turned off for a period of time after the induction; therefore, “turn-off” time periods of 3, 5–6, 9–10, 15, 20, and 25 min were tested. The amount of fluid infused up to those timepoints was evaluated, but none of those variables was strong enough for inclusion in the model.

### Illustrations

Figure 1B–E shows the ability of the final model to recreate the dependent variables with and without consideration of the covariates. Comparison of Fig. 1D and E illustrates the great importance of MAP to the urinary excretion.

**Fig. 2:**
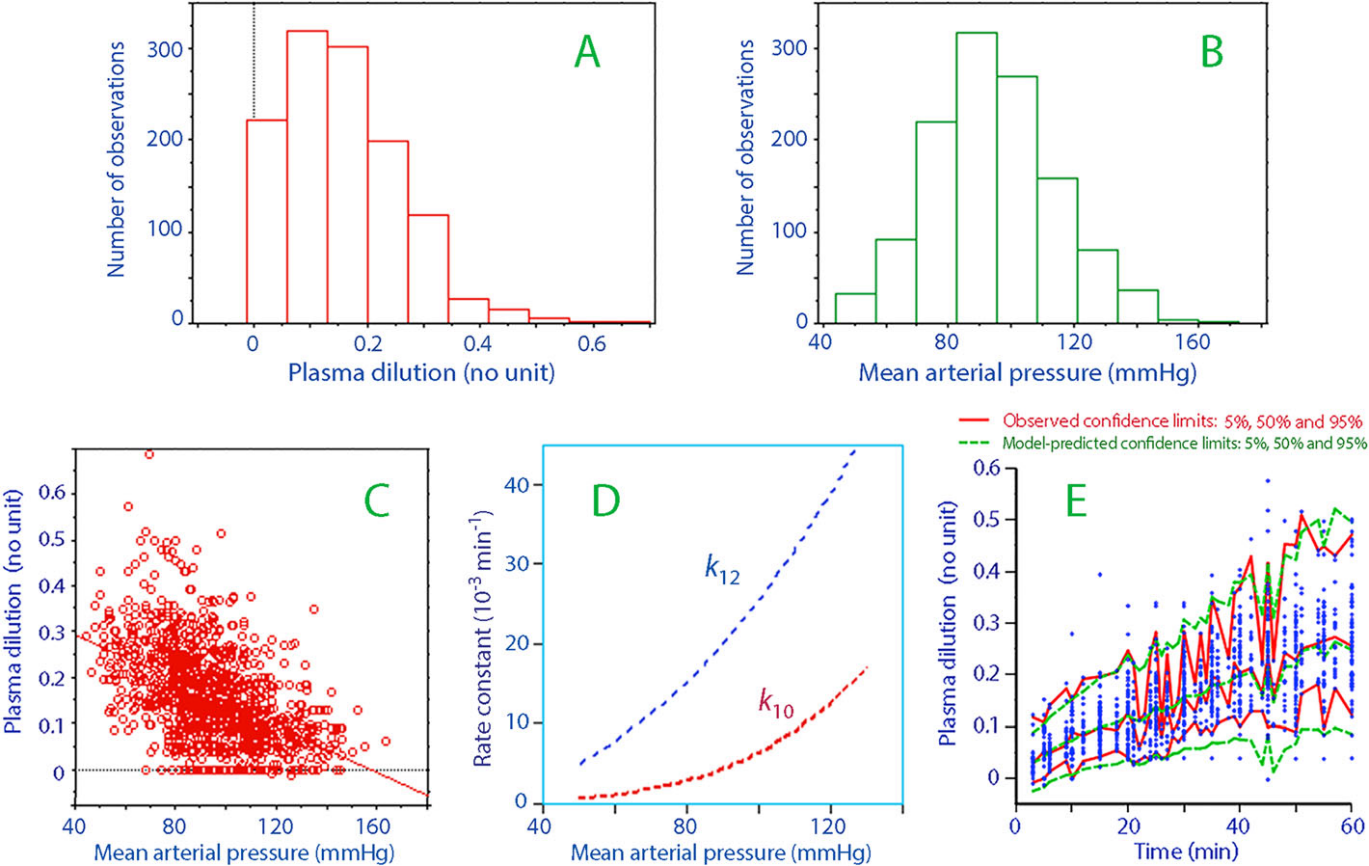
Distribution of observations of plasma dilution (**A**) and mean arterial pressures (**B**) and the relationship between these variables (**C**) throughout the study in all 76 patients, illustrating the limits for simulation by using the kinetic parameters. (**D**) Decrease in rate parameters for lower mean arterial pressures. (**E**) Predictive check showing the original data (blue points) with their confidence limits (red). The confidence limits based on 1000 simulations using the model parameters in the final model are superimposed (green lines). Hatched pattern is due to different sampling times. A small difference between observed and predicted confidence limits is a sign of good model performance. The difference between observed and predicted dilution averaged 0.03 (0.02) at the 5% level, −0.01 (0.05) at the 50% level, and −0.06 (0.06) dilution units at the 95% level

The distributions of the data on plasma dilution and MAP are given in Fig. 2A–B, and their relationship is shown in Fig. 2C.

The influence of MAP on *k*_12_ and *k*_10_ is shown graphically in Fig. 2D.

The performance of the model in the form of a predictive check based on 1000 simulations is illustrated in Fig. 2E.

### Secondary calculations

The model parameters in Table 3 were used to perform secondary calculations. These serve to illustrate the relevance of the kinetic data.

**Fig. 3:**
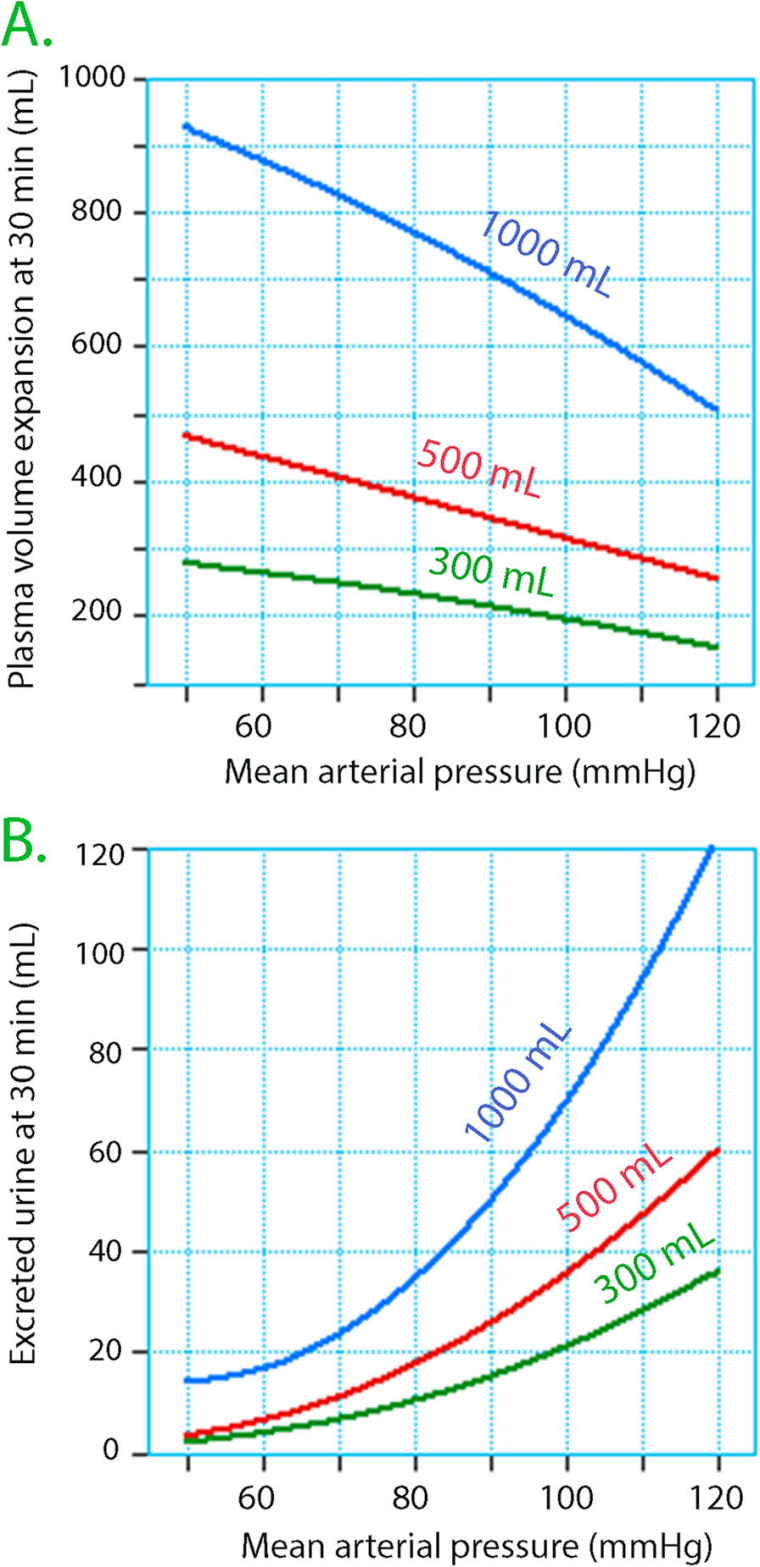
Simulations. Computer simulation showing (**A**) the plasma volume expansion and (**B**) the excreted urine volume at the end of a 30-min infusion of various volumes of crystalloid fluid. The parameter values from Table 3 were used

Figure 3 shows the magnitude of the fluid retention occurring when MAP decreases by entering the model parameters into a simulation program.

The rate constants can be used to calculate the distribution and elimination half-lives for different MAP. For example, the intravascular half-life T_1/2_ = natural logarithm of 2 (=0.693)/(*k*_10_ + *k*_12_) was 15 min at a MAP of 110 mmHg while being 80 min at a MAP of 60 mmHg. The corresponding values for the elimination T_1/2_, obtained as ln 2/*k*_10_, were 77 min and 737 min, respectively.

## Discussion

### Main findings

This report provides a mathematical description of what happens to the fluid distribution when anesthesia is induced in the middle of a continuous crystalloid infusion. The most apparent effect consists of a marked reduction in the rate constant that governs the distribution of infused fluid (*k*_12_) from the central fluid space (*V*_c_, the plasma) to the extravascular space. Fluid was distributed more slowly.

This change is probably an illustration of the classical Starling equation, which holds that the transcapillary exchange of fluid is determined by the balance between hydrostatic and oncotic forces across the capillary wall. A lowered MAP is likely to decrease the intravascular hydrostatic pressure. This, in turn, would reduce the capillary filtration because the interstitial hydrostatic pressure remains unchanged.

The elimination rate constant that describes urinary excretion (*k*_10_) also decreased, and to an even greater extent than was observed for the distribution. Both reductions were proportional in a non-linear fashion to the anesthesia-induced decrease in MAP, as shown in Fig. 2D and illustrated in Fig. 3.

### Clinical implications

The excessive intravascular accumulation of infused fluid during induction of anesthesia is apparently a consequence of the vasodilatation which degree is indicated by the decrease in MAP. There is limited evidence to suggest that the volume expansion would affect the arterial pressure except if vary large volumes are infused and, in particular, if the crystalloid fluid is replaced by a colloid (James and Dyer 2016). However, an often overlooked issue is that the volume expansion is mirrored by a reduction of the blood Hb level that will be much greater expected as a result of the vasodilatation, and this is relevant for perioperative medicine. Marked Hb changes occur even without hemorrhage when MAP is modulated by regional or general anesthesia. For example, the drop in Hb will decrease oxygen delivery if it is unmatched by an increase in the stroke volume. Furthermore, a pre-set Hb used as transfusion trigger will be reached more rapidly than is indicated by the surgical blood loss.

A dependency of the plasma volume expansion on MAP was observed previously in the papers underlying this work (Ewaldsson and Hahn 2001, 2005; Li et al. 2007). However, the present population kinetic analysis of the pooled data provides a more precise understanding of this relationship. The data presented here even allow simulations to be made that predict how variations in MAP and the amount and rate of infused fluid affect Hb. The following example, based on mass balance calculations (Ho et al., 2016), illustrates the influence of the reported fluid retention as compared to the conventional view of how fluid affects the blood Hb concentration. A widely cited relationship holds that infusion of 1 L of crystalloid increases the blood volume by 150 mL (Jacob et al., 2012), which would reduce blood Hb from 150 g/L to 144 g/L if the baseline blood volume is 5 L. Based on the relationships presented here, infusion of the same amount over 30 min during the induction of anesthesia would reduce Hb to 129 g/L if the MAP is 70 mmHg; i.e., 3.5 times more.

Some of this difference is not due to MAP but to the fact that crystalloid fluid shows a distribution function that requires 25–30 min for completion. In the example above, as much as 83% of the infused volume remains in the blood, and the patient will be close to being anuric if the anesthesia reduces MAP to 70 mmHg (Fig. 3). However, induction of general or regional anesthesia with unchanged MAP was still associated with a plasma volume expansion amounting to 50% at the end of a 30-min infusion of Ringer’s. This confirms previous findings in volunteers (Hahn 2010) and is 3 times greater than *after* the infusion is completed (Jacob et al. 2012).

The increased plasma volume expansion due to the MAP-dependent decrease in *k*_12_ is likely to remain until the intravascular hydrostatic pressure has increased sufficiently to reach a new Starling equilibrium, and this increase requires a vasoconstrictor, capillary refill, or additional infusion of fluid. The *k*_10_ value is known to remain low, despite adequate volume compensation, as long as MAP is low, but the normal value is resumed when the patient awakens from the anesthesia (Hahn 2020).

### Kinetic analysis

Several variables were evaluated that did not receive sufficient strength to be included in the kinetic model. For example, ephedrine administration had only an indirect effect via MAP on the kinetic parameters. Previous work has shown that buffered Ringer’s solution undergoes a more rapid turnover in young subjects than in aged subjects, but the current age span was probably too narrow to distinguish that relationship. No differences in fluid kinetics were found between patients who received spinal, epidural, or general anesthesia. The crude MAP, and not the change from baseline, governed the fluid kinetics, just as occurs during ongoing surgery (Hahn 2017).

A thorough evaluation was made to determine whether a normal *k*_12_ was resumed after a certain amount of fluid had been infused. However, no such “turning point” was found. The reason is probably that the infusion of 1.1 L of Ringer’s did not fill up the vasodilated cardiovascular system sufficiently to allow a resumption of the normal exchange of fluid with the extravascular space. In a previous study, this “turning point” was reached when 16.6 mL/kg of Ringer’s (1.25 L) had been administered (Hahn and Nemme 2020). This probably corresponds to the anesthesia-induced expansion of the part of the blood volume that is sometimes called “unstressed” and which denotes the amount of venous blood that does not increase the transmural pressure (Gelman 2008).

### Current versus previous models

The excessive accumulation of infused Ringer’s solution during the onset of regional anesthesia was first studied by linear regression and reported in the early 1990s (Hahn 1992). A later study showed that the decrease in MAP appears a few minutes before the increased hemodilution, and this finding clarified the order of events (Drobin and Hahn 1996). Subsequent analyses of the fluid kinetics during induction of anesthesia applied a clearance version with a single inter-compartmental clearance parameter (Li et al. 2007), but this is problematic because the Starling forces are changed in the middle of the experiment. The present population kinetic model separates the flows in and out of the plasma volume and shows clearly that no return of fluid to the plasma occurs during the onset of anesthesia as long as fluid loading is ongoing.

The current model also uses micro-constants instead of clearances, which makes it independent of plasma volume and body fluid volumes. The micro-constant model detects a “wall” between a central space, where fluid equilibrates very rapidly with the site of infusion, and a more remote peripheral space. The space with this fast equilibration is very likely to represent the plasma volume contained in blood vessels that are allowed to expand. The exchange of infused fluid between these two body fluid spaces is determined by rate constants (*k*_12_ and *k*_21_). The volume of the infused fluid residing in the two body fluid spaces is obtained directly in the micro-constant model, while their dilution must be multiplied by the volume of distribution to obtain volume expansion in the clearance model.

### Limitations

The limitations of the present study include that the data were obtained from three previously published works, although all three used a similar protocol and sampling had been performed in the same way.

The fairly low values of *k*_12_ and *k*_10_ before the induction may reflect preoperative anxiety, which has been observed in adults and children alike (Li et al. 2009). Higher values would be expected if the fluid had been infused in volunteers not scheduled for surgery (Hahn 2020).

The strength of this study is that a modern and robust kinetic approach was used that allows simultaneous analysis of all studied patients, as well as comparisons of several different covariances that could potentially influence the fluid kinetics.

## Conclusion

The induction of regional and general anesthesia was followed by a MAP-dependent decrease in both the distribution and elimination of an infused crystalloid fluid. Both changes markedly increased the hemodilution, which might become large enough to affect oxygen delivery and distort schemes for deliberate hemodilution and estimates of allowable blood loss.

## Supplementary Information

**Additional file 1.**

## Data Availability

The data used for the kinetic analysis are available as Supplementary file 1.

## Abbreviations

Hb: Hemoglobin
i.v.: Intravenous
*k*_12_: Rate constant for fluid passing from *v*_c_ to the extravascular fluid space
*k*_21_: Rate constant for fluid passing from the extravascular space to *v*_c_
*k*_10_: Rate constant for fluid eliminated by urinary excretion
*k*_b_: Rate constant for eliminated fluid not recovered as urine
MAP: Mean arterial pressure
NLME: Non-linear mixed effects
R_o_: Infusion rate
*V*_c_, *v*_c_: Sizes of central body fluid space at baseline and during fluid therapy, respectively
T_1/2_: Half-life
